# Psychosocial factors and Burnout Syndrome among mental health professionals*

**DOI:** 10.1590/1518-8345.4175.3336

**Published:** 2020-08-31

**Authors:** Amanda Sorce Moreira, Sergio Roberto de Lucca

**Affiliations:** 1 Universidade Estadual de Campinas, Faculdade de Ciências Médicas, Campinas, SP, Brazil.

**Keywords:** Occupational Stress, Burnout, Professional, Mental Health, Health Personnel, Professional Autonomy, Social Support, Estresse Ocupacional, Esgotamento Profissional, Saúde Mental, Profissionais da Saúde, Autonomia Profissional, Apoio Social, Estrés Laboral, Agotamiento Profesional, Salud Mental, Personal de Salud, Autonomía Profesional, Apoyo Social

## Abstract

**Objective:**

to identify biopsychosocial factors at work associated with the Burnout Syndrome in mental health professionals.

**Method:**

a cross-sectional study with a quantitative approach conducted with a sample of 293 mental health service workers from the public network of a municipality in the inland of the state of São Paulo, Brazil. An instrument composed of three self-administered questionnaires was applied, namely: biosocial data form, the Job Stress Scale (JSS) and the Maslach Burnout Inventory (MBI- HSS). The data were analyzed through the application of the Chi-squared and logistic regression tests, with the adoption of a 5% significance level.

**Results:**

Burnout Syndrome prevalence was 7% with a predominance of nursing professionals and was associated with the work sector, the use of psychotropic drugs, low satisfaction with the manager and with the low control over the work activity. Among the professionals with Burnout Syndrome, twelve performed functions considered of high wear, six performed passive work and two were in low wear activity.

**Conclusion:**

low control was the main psychosocial factor at work associated with Burnout Syndrome, making it necessary to develop actions that promote worker autonomy and improve the management of stress-triggering psychosocial factors.

## Abstract (pt)

**Objetivo:**

identificar os fatores biopsicossociais no trabalho associados à Síndrome de *Burnout* em profissionais da saúde mental.

**Método:**

estudo transversal de abordagem quantitativa de uma amostra de 293 trabalhadores dos serviços de saúde de mental da rede pública de um município, no interior do estado de São Paulo, Brasil. Foi aplicado um instrumento composto por três questionários de autopreenchimento: formulário com dados biossociais, a escala *Job Stress Scale* (JSS) e o *Maslach Burnout Inventory* (MBI- HSS). Os dados foram analisados por meio da aplicação dos testes Qui-Quadrado e regressão logística, adotando-se um nível de significância de 5%.

**Resultados:**

a prevalência da Síndrome de *Burnout* foi de 7% com predomínio de profissionais da enfermagem, estando associada ao setor de trabalho, ao uso de psicofármacos, à baixa satisfação com a chefia e ao baixo controle sobre a atividade de trabalho. Entre os profissionais com Síndrome de *Burnout*, doze desempenhavam funções consideradas de alto desgaste, seis exerciam trabalho passivo e dois estavam em atividade de baixo desgaste.

**Conclusão:**

o baixo controle foi o principal fator psicossocial no trabalho associado à Síndrome de *Burnout*, tornando-se necessário o desenvolvimento de ações que promovam a autonomia do trabalhador e a melhoria da gestão dos fatores psicossociais desencadeantes de estresse.

## Abstract (es)

**Objetivo:**

identificar los factores biopsicosociales en el trabajo asociados con el *Síndrome de Burnout* (SB) en profesionales de la salud mental.

**Método:**

estudio transversal con enfoque cuantitativo de una muestra de 293 trabajadores de servicios de salud mental en la red pública de un municipio del interior del estado de São Paulo, Brasil. Se aplicó un instrumento compuesto por tres cuestionarios autoadministrados: un formulario con datos biosociales, el Cuestionario de Estrés Laboral (JSS) y el Inventario de Maslach (MBI-HSS). Los datos se analizaron mediante la aplicación de las pruebas de Chi-cuadrado y la regresión logística, adoptando un nivel de significación del 5%.

**Resultados:**

la prevalencia de *Síndrome de Burnout* fue del 7% con un predominio de profesionales de enfermería, hallándose asociado a factores como el entorno laboral, uso de drogas psicotrópicas, baja satisfacción con el jefe y bajo control sobre la actividad laboral. Entre los profesionales con *Síndrome de Burnout*, doce desempeñaron funciones consideradas de alto desgaste, seis cumplían tareas pasivas y dos realizaban una actividad de bajo desgaste.

**Conclusión:**

el bajo control fue el principal factor psicosocial asociado con el *Síndrome de Burnout* en el trabajo de los profesionales, por lo que es necesario desarrollar acciones que promuevan la autonomía del trabajador y el mejoramiento en la gestión de los factores psicosociales que desencadenan el estrés.

## Introduction

The inclusion of the Unified Health System (*Sistema Único de Saúde* - SUS - in Portuguese language) principles in the 1988 Constitution broadened perspectives on the Psychiatric Reform movement, resulting in a network of mental health care and assistance capable of offering home care, outpatient and hospital care, Psychosocial Care Centers (*Centros de Atenção Psicossocial*, - CAPS - in Portuguese language) and Home Therapeutic Services (*Serviços Residenciais Terapêuticos* - SRT - in Portuguese language)^(^ 1 ^)^.

The work of the professionals in these services is complex and depends on the articulation of multidisciplinary teams, evolving different knowledge, practices and experiences^(^ 2 ^)^. These workers experience intense and antagonistic feelings, often conflicting, in addition to the high resoluteness charges, work overload and precarious working conditions, which may compromise their health^(^ 3 ^)^.

In this context, chronic exposure to stressors in the work environment has as one of the negative outcomes the Burnout Syndrome (BS). The syndrome mainly affects professionals in services dealing directly with people and results from the interaction between psychosocial factors and individual characteristics^(^ 4 ^)^.

In addition to the peculiarities of mental health work, professionals in these services are more exposed to situations of violence because they deal with patients in acute crisis and who require help and permanent supervision^(^ 5 ^)^.

Psychosocial factors, work-related stress, violence and (moral and/or sexual) harassment are, currently, the greatest challenges in the field of occupational health and safety^(^ 6 ^)^. The Psychosocial factors at work (*Fatores Psicossociais no Trabalho* - FPT- in Portuguese language) arise from the interactions between the worker and the work environment, working conditions and organization, work demands and the degree of autonomy over activities and they can influence health, performance and job satisfaction^(^ 7 ^)^.

Among the instruments for evaluating these factors, it is the Demand-Control-Social Support model, which checks the relationship between psychological demands and the degree of control in work activities with potential for stress and exhaustion^(^ 8 ^)^. This model establishes that work processes with high demands and low control have a greater potential for illness, while social support would play a mediating role in this relationship^(^ 9 ^)^.

Studies on FTPs and BS in mental health service’s professionals are still scarce in the literature. Thus, considering the recognized emotional burden and stress in these services, this study sought to identify the biopsychosocial factors at work associated with BS in mental health professionals.

## Method

An epidemiological, cross-sectional, and descriptive study with a quantitative approach conducted with a sample of professionals from a mental health service provided by a public institution, located in Casa Branca, a municipality of the inland of São Paulo, Brazil, in the period from January to February 2019.

The criteria for choosing the institution and sample selection were based on the particular characteristics of the teams regarding the direct and indirect care given to patients with mental disorders, previously, observed by the researchers during the non-participating observation stage in the three work shifts, in order to know and contextualize the work particularities of the respective services.

The institution is a regional reference in mental health care for eleven municipalities, offering long-stay admission services with a female, male, and geriatric quarters, outpatient care, and short-term admission through the type III Center for Psychosocial Care (CAPS III), and thirty homes that comprise the Therapeutic Residency Services (*Serviços de Residências Terapêuticas* - SRT - in Portuguese language). All care services are full time and professionals work in three fixed shifts (morning, afternoon and evening), while administrative professionals work from Monday to Friday, from 7:00 a.m. to 2:00 p.m. The institution had 547 professionals, 405 from the care sector and 142 from the administrative sector, including managers.

As inclusion criteria for the survey, all care and administrative professionals, who had been active in the institution for more than six months, were included. Those who were on days off, holidays, away on medical or maternity leave, who were not found after three attempts of contact and those who filled in incompletely the data collection instrument were excluded.

The sampling process was carried out using the convenience non-probabilistic method, resulting in a sample of 293 professionals, since 58 participants did not meet the inclusion criteria, 23 refused to participate in the survey and 173 did not return the instrument.

The data were collected using a self-completion instrument, composed of three questionnaires: the biosocial questionnaire, prepared by the authors and based on relevant studies on the topic^(^ 8 ^,^ 10 ^-^ 11 ^)^, the Job Stress Scale (JSS), a short version of the Demand and Control model and the Maslach Burnout Inventory - Human Services Survey (MBI-HSS). The instrument was pre-tested by the researchers in a group of ten professionals from different shifts.

The biosocial questionnaire was composed of 27 multiple-choice questions on gender, marital status, age, race/color, disabilities, dependents, schooling, place and sector of work, position/function, working time in this institution and in the current position, working hours, shifts, overtime, the occurrence of harms to health (accidents and occupational illness) and moral and/or sexual harassment, use of psychotropic, alcohol and other drugs, satisfaction with the work, the institution, the manager and co-workers, and what this employee, would possibly, change in this service.

Questions about position/function, accidents at work, and work-related illnesses were semi-open so that participants could report their answers. In order to evaluate the perception of satisfaction, we chose not to apply specific instruments, because they do not match the objectives of the research. The participants should mark zero, five, or ten for low, moderate, and high satisfaction respectively. In addition, on the question of suggested changes, participants could point out more than one alternative and, also, describe other changes that were not listed by the researchers.

The MBI-HSS is a three-dimensional questionnaire intended for health professionals^(^ 12 ^)^, translated into and validated for Portuguese^(^ 11 ^)^, consisting of 22 multiple-choice questions, 9 concerning the dimension of emotional exhaustion (EE), 5 concerning depersonalization (DE) and 8 concerning low personal fulfillment (PF)^(^ 11 ^-^ 12 ^)^. Responses vary according to their frequency of occurrence and to each one, the following points are assigned: “never”, gets zero, “a few times a year”, 1 point, “a few times a month”, 2 points, “a few times a week”, 3 points, and “daily”, 4 points. At the end of the sum of each dimension, the score is estimated by calculating the quartiles^(^ 11 ^)^. Cases with high EE and DE and low PF, concomitantly are considered as indicative of Burnout^(^ 11 ^-^ 12 ^)^.

The Job Stress Scale (JSS) or “Swedish Demand-Control-Social-Support Scale” is a three-dimensional Likert-type scale that assesses psychosocial factors and risk of stress in work activities^(^ 8 ^,^ 13 ^)^, translated into and validated for Portuguese^(^ 8 ^)^, composed of 17 questions, of which 5 are about psychological demand, 6 about task control and 6 about social support. For the demand and control dimensions, the responses vary according to the frequency of occurrence, and the following points are assigned to each one: “often”, 4 points, “sometimes”, 3 points, “rarely” gets 2 points, and “never/almost never”, 1 point. In the social support dimension, for the statement “I totally agree”, 4 points are given, “I agree more than I disagree”, 3 points, “I disagree more than I agree”, 2 points, and “I totally disagree”, 1 point. At the end of the sum of the three dimensions, the median is calculated; this analysis allows working conditions to be classified in the quadrants of the model: active work, passive work, low demand work, and high demand work^(^ 8 ^)^.

The collected data were analyzed by means of descriptive statistics and presented in the form of frequency tables with absolute (n) and percentage (%) values. All independent variables were tested, both for the occurrence of BS and for comparison between sectors. The Chi-squared test was used for comparison between the sectors and, for the Odds Ratio estimate for the occurrence of Burnout, the simple and multiple Logistic Regression analyses, adjusted by stepwise selection criteria, were used. The Statistical Analysis System (SAS) program, version 9.4, was used.

All ethical principles of research with human beings were respected and the study was submitted to and approved by the local Ethics Research Committee (CEP), under the following opinion CAAE No.: 93599118.0.0000.5404, on September 26^th^, 2018.

## Results

In this study, there was a predominance of female workers (64.8%), aged 45 years old or older (66.3%), who had a partner (62.5%), children (59.4%) and no dependents (74.7%). There were no differences between the percentages of participants with basic education level (Elementary and Secondary - 50.9%) and higher education level (Higher and Graduation - 49.1%).

Most of the participants (87.4%) worked in the long-term service, in care activities, distributed among the positions of auxiliary and nursing technician (150), nurse (13), doctor (5), dentist (1), health and therapeutic function assistant (16) and health care technical agents (psychologist, social worker, occupational therapist, physiotherapist, pharmacist and pharmacy technician).

Among those who performed exclusively administrative activities (29.7%), 12 held the posts of health officers, seven administrative officers, eight chief of health, and two health directors. Some other 55 professionals in the function of general services assistant, worked in the laundry, kitchen, janitorial, warehouse, electric maintenance, agriculture/gardening, and three drivers.

There was a predominance of professionals working in the morning (60.8%), due to the working hours of both (care and administrative) sectors, with working hours of up to 30 hours *per* week (91.8%). The professionals who worked overtime, mostly, worked in the night shift. About one third of the participants reported having a second job.

The alcohol consumption was self-reported by 58% of the subjects and 28% reported the use of psychotropic and illicit drugs. Regarding work-related diseases, 53 professionals reported the occurrence of Musculoskeletal System diseases and 33 participants reported Mental and Behavioral Disorders. Among the professionals in the assistance, moral and/or sexual harassment was reported by 32% of the participants.

As for job satisfaction, 48% of the participants were satisfied with their co-workers and management, while 62.4% expressed a moderate satisfaction degree with the institution.

Among the factors of dissatisfaction, the subjects reported the lack of career planning, bonuses and awards, inadequate ergonomic conditions and difficulties in relationships between team members.

**Figure 1:**
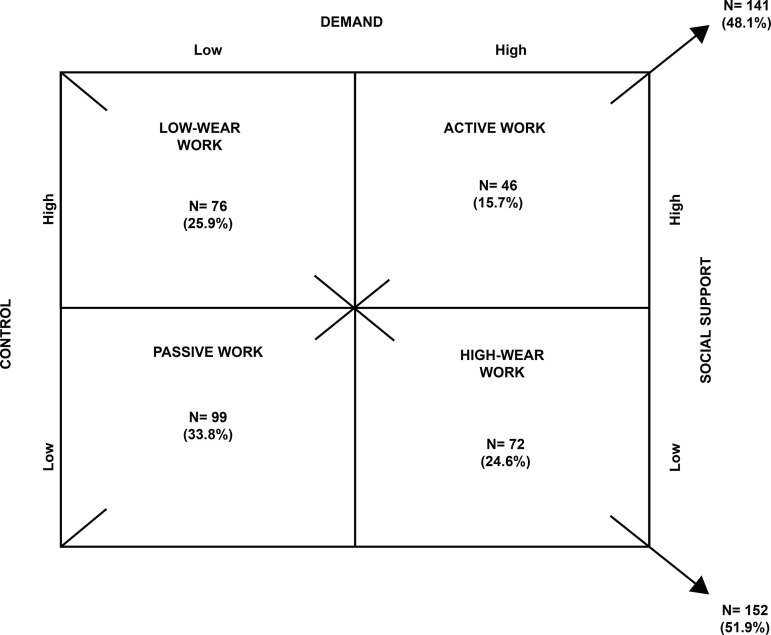
Distribution of the workers (n=293) according to the Demand-Control-Social Support Model. Casa Branca, SP, Brazil, 2019

Regarding the psychosocial factors, considering the means and medians obtained in the psychological demands and control dimensions, it was possible to classify and distribute the work activities in the Demand-Control model, as described in Figure 1.

The data in Figure 1 showed that 33.8% of the participants worked in activities with low demands and control and low social support (passive work). In relation to the administrative workers, 27.6% performed high-wear work activities, 26.4% passive work, 25.3% active work, and 20.7% low-wear work. On the other hand, of the care professionals, 36.9% carry out passive work activities, 28.1% low-wear work, 23.3% high-wear work, and 11.7% active work. Social support was more prevalent among administrative workers (62%), compared to care workers (42.2%). The “psychological demands” (p=0.004) and “social support” (p=0.001) dimensions showed statistical associations between the sectors wherein these professionals were inserted.

The MBI descriptive analysis, obtained through the quartile distribution, showed that most workers in these mental health services had moderate EE, low DE, and moderate PF, as described in Table 1.

**Table 1:** Distribution of the workers' frequency (n=293) according to the cut-off points of the MBI- HSS dimensions' quartiles. Casa Branca, SP, Brazil, 2019 (n=293)

Dimension	Cut-off points	N	(%)
Emotional Exhaustion (EE) - Low - Moderate - High	≤ 7 8 - 19 ≥ 20	74 141 78	25.26 48.12 26.62
Depersonalization (DE) - Low - Moderate - High	= 0 1 - 4 ≥ 5	140 68 85	47.78 23.21 29.01
Personal Fulfillment (PF) - Low - Moderate - High	≤ 23 24 - 30 ≥ 31	88 119 86	30.03 40.61 29.35

The prevalence of BS among the professionals who presented, concomitantly, high EE, high DE, and low PF was 7% (20 professionals), with predominance among the care workers (p=0.039), in the function of nursing assistants (p=0.039).

It was verified that most administrative workers had moderate EE and low PF. The percentages of these professionals with low (40.2%) and high (39%) depersonalization were very close. Among the care professionals, the majority had moderate EE and PF and low DE. Only the EE dimension did not show a statistical difference between sectors (DE: p=0.046; PF: p=0.003).

The study variables associated with the Burnout Syndrome (p<0.05) and with the respective odds ratio for the illness of these mental health service professionals are described in Table 2.

**Table 2:** Gross Odds Ratio of the Burnout Syndrome in mental health professionals (n=293) according to biosocial and work characteristics and the psychosocial factors at work. Casa Branca, SP, Brazil, 2019

Variable	Category	p-value	OR	95% Confidence Interval
Schooling	Elementary and High x Higher	0.044	2.92	1.02; 8.34
Use of psychotropic drug	Yes x No	0.028	2.79	1.16; 6.98
Time in the sector	≤ 5 years x > 5 years	0.028	3.02	1.12; 8.09
Accident at work: Aggression	Yes x No	0.029	2.96	1.11; 7.85
Satisfaction in relation to: - Work - Institution - Co-worker - Management	High x Low High x Low High x Low High x Low	0.010 0.038 0.043 0.007	5.00 3.16 4.76 12.00	1.46; 17.09 1.06; 9.41 1.04; 21.7 3.07; 46.88
Desire for changes: Management profile	Yes x No	0.045	2.56	1.01; 6.46
Control	Greater x Minor	0.009	7.05	1.60; 31.01
Social Support	Greater x Minor	0.014	4.02	1.31; 12.36

The multiple analysis confirmed the association of the following variables: schooling [OR=5.50; 95% CI (1.29; 23.33)], work sector [OR=18.37; 95% CI (3.85;87.48)], use of de psychotropic [OR=5.21; 95% CI (1.38; 19.98)], satisfaction with the management [OR=40.46; 95% CI (5.63; 29.05)] and control over work activities [OR=11.71; 95% CI (2.50; 11.2)].

For the analysis of the association of the JSS and MBI scales’ dimensions, the low demand, high control and high social support variables were used as a reference. Table 3 describes such associations and the gross odds ratios for the occurrence of high EE, high DE, and low PF.

**Table 3:** Gross Odds Ratio between the dimensions from the stress scale at work with high Emotional Exhaustion (EE), high Depersonalization (DE), and low Personal Fulfilment (PF). Casa Branca, SP, Brazil, 2019

**Dimension**	**High Emotional Exhaustion (EE)**		
	**p-value**	**Gross OR**	**95% Confidence Interval**
Demand	0.001	10.22	4.64; 22.52
Control	0.037	2.01	1.04; 3.14
Social Support	0.001	4.43	2.24; 8.76
	**High Depersonalization (DE)**		
	**p-value**	**Gross OR**	**95% Confidence Interval**
Demand	0.002	3.24	1.84; 5.70
Control	0.793	1.14	0.66; 1.99
Social Support	0.407	1.41	0.82; 2.43
	**Low Personal Fulfillment (PF)**		
	**p-value**	**Gross OR**	**95% Confidence Interval**
Demand	0.044	2.18	1.78; 4.04
Control	0.006 0.022	1.90 2.34	1.01; 3.57
Social Support	0.035	2.20	1.02; 4.04

In the multiple analysis, there has been association between high DE and work demand [OR=3.43; 95% CI (1.87; 6.30)], while low PF was associated with control [OR=2.34; 95% CI (1.17; 4.67)] and social support [OR=2.77; 95% CI (1.40; 5.46)].

**Figure 2:**
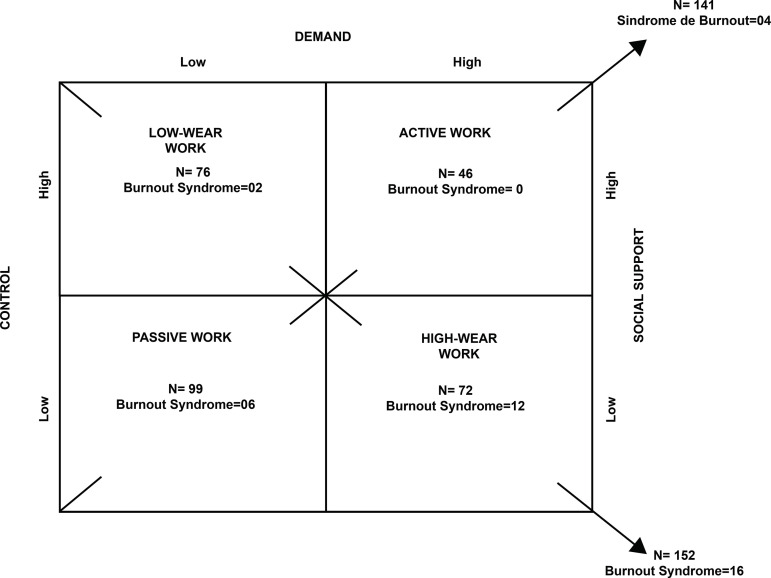
Distribution of the number of professionals (n=293) with Burnout Syndrome according to the classification of work and based on the Demand-Control-Social Support model. Casa Branca, SP, Brazil, 2019

Figure 2 illustrates the distribution of the number of professionals with Burnout in the respective quadrants of the Demand-Control-Social Support model.

## Discussion

Identifying the main psychosocial factors that trigger stress at work among mental health professionals is crucial to understand the stress and BS triggers in these services since the diagnosis of the situation can help in the development of strategies to promote and protect workers’ health in the context of work organization.

Although there is no consensus in the literature on the interpretation of results for determining the prevalence of the Burnout Syndrome, which varies between 5.0 and 55.3%, depending on the analysis criteria adopted, a percentage of 7% was observed in this population, proportional to that found in studies with health professional^(^ 14 ^)^.

In the isolated analysis of the dimensions, 26.6% of the participants presented high emotional exhaustion, 29.0% high depersonalization and 30% low professional fulfillment. These results were similar to other studies that resulted in the prevalence of high emotional exhaustion between 17.2% and 53.3%; high depersonalization (DE) between 17.8% and 93.7% and low personal fulfillment (PF) between 18.8% and 93.7%^(^ 14 ^)^.

Biosocial characteristics do not always show an association with the three dimensions that conceptualize the BS, but they may present association with some dimension. Research with professionals from a psychiatric hospital in Australia showed that Burnout was associated with gender (female vulnerability), age (under 45), and sector of work (contact with the patient)^(^ 15 ^-^ 16 ^)^. In fact, the care process peculiar aspects contribute to the feminization of some occupations and professions, as observed in the health sector^(^ 15 ^)^.

The low turnover and stability of professionals in the public mental health institution surveyed may justify the higher prevalence of the syndrome among those over 45 years old found in this study.

As evidenced by other authors, the shorter time in the sector was another factor associated with Burnout. Workers with less time in the institution or sector are more likely to develop the syndrome due to the difficulties of insertion into the group, feeling of insecurity, job instability associated with the need for acceptance^(^ 17 ^)^. Workers at the beginning of their career are more likely to manifest the syndrome because they perceive higher the interpersonal demands at work^(^ 18 ^)^.

The relationship between Burnout Syndrome and psychic diseases is controversial, but researchers consider the syndrome as a risk factor for the consumption of antidepressants^(^ 19 ^)^. This study showed an association between the consumption of psychotropic drugs and professional exhaustion, while having mental and behavioral disorders resulting from work was associated with high emotional exhaustion^(^ 20 ^)^.

Professionals who suffer physical aggression from patients are more vulnerable to emotional exhaustion and also to BS^(^ 21 ^)^. In addition to professional exhaustion, professionals who have already suffered aggression also have greater dissatisfaction, work overload and low commitment, in addition to increasing work turnover^(^ 22 ^)^.

The association between job satisfaction and Burnout is scientifically proven and it suggests that both low satisfaction can cause work exhaustion and as burnout can be the cause of dissatisfaction^(^ 23 ^-^ 24 ^)^. In this study, the low satisfaction or indifference of professionals in relation to the institution, the work, the co-workers and the management proved to be associated with BS and some of its dimensions.

Professionals with worse Burnout psychic symptoms have lower job satisfaction, specially, the younger ones^(^ 24 ^)^. A survey showed that multi-professional teams in a psychiatric hospital were dissatisfied with the lack of autonomy and participation in decision-making within the service regarding financial issues and benefits, and were moderately overloaded^(^ 25 ^)^.

Dissatisfaction with management, with the lack of professional growth and benefits can increase symptoms of emotional exhaustion, depersonalization and, consequently, of Burnout^(^ 25 ^-^ 27 ^)^. These factors also presented an association with the syndrome, resembling a study with psychiatric professionals in Greece^(^ 27 ^)^.

It is evident that each job has its own characteristics and that each worker understands these factors in a subjective way, and it is this perception that modulates the levels of Burnout^(^ 26 ^)^.

The types of tasks influence the worker’s perception of physical and psychological demands and of the work process, with direct patient care being recognized as the most demanding in health services and may be perceived as rewarding or not. The task of dealing with documents, on the other hand, is less rewarding, although professionals recognize it as a work of less demand and greater control^(^ 28 ^)^.

High work demands, low control over the work process and lack of social support significantly influence satisfaction^(^ 21 ^,^ 29 ^-^ 30 ^)^. A study on the interaction of the psychosocial factors with the Burnout dimensions showed that the psychological demand was the one most related to emotional exhaustion, while control over the work process was more associated with depersonalization and personal fulfillment^(^ 31 ^)^. Besides, the authors also found that the interaction between demand, control and social support was significant for the occurrence of emotional exhaustion and depersonalization^(^ 31 ^)^.

The predominance of BS among professionals who perform high-wear activities coincides with those of other researches, which shows that Burnout is related to the lack of autonomy, high workloads, the performance of multiple functions and low social support^(^ 32 ^-^ 33 ^)^. Other studies indicate that both high psychological demands at work and low control over work activities are often associated with Burnout; however, over time, high demands seem to be more associated with the syndrome than with low control over the work process^(^ 34 ^)^.

Emotional exhaustion is the dimension of professional exhaustion most strongly influenced by work psychosocial factors and the work demand is the dimension of stress most related to this factor^(^ 26 ^,^ 35 ^-^ 36 ^)^. The associations between emotional exhaustion, work demand, and low satisfaction suggest that the worker with high exhaustion tends to perceive higher psychological demand and fatigue^(^ 37 ^)^. The data observed among administrative professionals in this study are similar to such statement. However, although there is a difference between the levels of work demands and of Burnout, between the care and administrative sectors, the severity of these factors differ little, even when health professionals tend to report a greater workload^(^ 37 ^)^.

Research on the relationship between work demands and low personal fulfillment is still scarce^(^ 38 ^)^; however, in the aforementioned survey, there was an association between them, with the odds ratio estimated at 1.53.

Studies performed to estimate Burnout risk, due to exposure to psychosocial factors at work showed that, besides the low control being associated with the syndrome, the chances of having high emotional exhaustion in these situations were 1.63^(^ 38 ^)^, similar to the data obtained in this research.

Autonomy is considered a mediator of psychological wear^(^ 39 ^)^. The lower the control over the work process, the greater the emotional exhaustion, independently from the work load^(^ 22 ^)^, that is, having more control over the work protects the worker from exhaustion.

Some studies indicate that low control and low social support increase the chances of having BS^(^ 5 ^,^ 21 ^,^ 31 ^,^ 36 ^)^. However, acting only on these factors may not diminish the chances of illness, because it is not enough just to improve social interaction at work and promote greater autonomy; it is necessary, fundamentally, to reduce the high psychological demands to which these professionals are subjected for the effective reduction in the work environment^(^ 40 ^)^.

We observe that the worker exposed to high-wear situations does not always have greater exhaustion since the three dimensions of the syndrome relate differently to the main psychosocial factors (demand, control, and social support)^(^ 36 ^)^. Another study also found an association of the emotional exhaustion and personal fulfillment dimensions with all the psychosocial dimensions, as well as the association of depersonalization with the psychological demand and social support dimensions^(^ 41 ^)^.

Most studies found no association between low social support and depersonalization, but we believe that high social support reduces the chances of manifesting such a dimension^(^ 38 ^)^.

Professionals in active working conditions and high social support prove to be more personally fulfilled than those in the same working conditions, despite the low social support^(^ 36 ^)^. Among the workers in this study, it was observed that the administrative professionals had greater social support while care workers reported low support. This situation suggests that in the care sector there were more interpersonal problems than in the administrative sector, reinforcing the idea that the profile of the management, colleagues and work organization reflects on the quality of social support and increases the chances of having BS.

The distribution of workers in this study regarding the type of work, considering the level of psychological demand and control over the work process, was equivalent to a study on the evaluation of occupational stress in care and administrative workers at a university. Both showed that most workers were exposed to low demand and low control situations, configuring it passive work, followed by low-wear work^(^ 42 ^)^.

As already observed in another research^(^ 43 ^)^, both care and administrative sectors can be classified as passive work. The prevalence of mental health service’s care workers in the passive work scenario can be explained by the fact that most of them are nursing auxiliaries and technicians, not performing managerial functions, which supposedly offer greater control over the work process^(^ 44 ^)^.

A survey pointed out the predominance of technical-administrative workers in situations of passive work, followed by low demand work, high demand work, and active work^(^ 45 ^)^. In this study, the predominance of Burnout has occurred in high-wear situations. This divergence between research may be associated with distinct institutional characteristics since one occurred in a university and the other in a public mental health care institution. However, the social support factor presented a similar distribution in both pieces of research and the technical-administrative professionals received high social support^(^ 45 ^)^.

The predominance of care professionals in work situations is common in the literature^(^ 46 ^)^, although there is evidence that these workers may also be exposed to low-wear situations^(^ 40 ^,^ 47 ^)^ and active work^(^ 48 ^)^. However, the percentage of workers exposed to high wear between the studies were similar^(^ 40 ^,^ 46 ^-^ 48 ^)^.

In this survey, two professionals who performed managerial functions fell ill in a situation of low wear, results similar to those of a survey with health managers, in which a great concentration of these professionals was observed in these same work situations^(^ 44 ^)^.

Passive work is considered the second biggest cause of illness, because in these situations the professional may manifest disinterest in the work and lose skills^(^ 46 ^)^. As well as in another study, this research also found cases of Burnout in care professionals exposed to passive work^(^ 48 ^)^. Social support serves as a buffer against illness in passive work situations since, working in situations of low control and low demand, but receiving support, reduces the chances of having BS^(^ 47 ^)^.

Exposure to high-wear work associated with low social support can result in high exhaustion^(^ 31 ^)^. Unlike this study, some researchers found a higher prevalence of nurses in situations of low control and high demands arising from work in intensive care units^(^ 49 ^)^.

On the other hand, the highest prevalence of BS in mental health service workers was in high-wear work situations, similar to other studies that also related professional burnout to high-wear activities^(^ 48 ^-^ 49 ^)^.

Thus, this reinforces the need to implement permanent strategies for identifying psychosocial factors to reduce work stress, in order to minimize the risk of professional burnout, to avoid new cases and the worsening of the sick who continue working^(^ 50 ^)^.

The study was unprecedented in Brazil since it took place in a public mental health institution, provider of several services (long-term admission, CAPS III and SRT), covering multiple (administrative and care) sectors and positions. Also, this research contributed to better describe the relationships in the subgroups investigated with the Burnout Syndrome and the psychosocial factors with the potential to trigger stress at work.

In cross-sectional studies, data on exposure and outcome are collected simultaneously, which makes inferences on causality impossible, although there is significant evidence to support such association. Since this is a convenience sample and considering the heterogeneity of this population, the results of this study present limitations regarding its potential for generalization and comparison, due to the peculiarities of each institution and biopsychosocial and occupational factors. In addition, the representativeness of the sample and the high non-response rate (non-response bias) may overestimate or underestimate the results. Other probable limitations were the memory bias due to the instrument’s self-reporting and the healthy worker bias, common in this type of research.

## Conclusion

The present study identified a 7% prevalence of the Burnout Syndrome among mental health service professionals, mainly among female nursing professionals (auxiliaries and technicians), older than 45 years of age, and with less time working in the institution and in the sector.

There were associations of the syndrome with the work sector, the use of psychotropic drugs, low satisfaction with the manager, and low control at work. Most of the Burnout cases ran among the professionals in work situations called high-wear work, characterized by high work demand and low autonomy.

The work activities with high psychological demands and low control over the tasks were the main psychosocial factors associated with the Burnout Syndrome. Thus, more detailed studies are needed, with a deepening of individual and organizational factors, to develop ways of working that make it possible to minimize the impacts on the health of workers in mental health services.
